# Life expectancy in individuals with type 1, type 2 diabetes and without diabetes: a systematic review and meta-analysis

**DOI:** 10.3389/fendo.2025.1704277

**Published:** 2025-11-13

**Authors:** Yasmin Ezzatvar, José Francisco López-Gil, Rodrigo Yáñez-Sepúlveda, Juan Hurtado-Almonacid, Antonio García-Hermoso

**Affiliations:** 1 Lifestyle Factors with Impact on Ageing and Overall Health (LAH) Research Group, Department of Nursing, University of València, Valencia, Spain; 2 Vicerrectoría de Investigación y Postgrado, Universidad de Los Lagos, Osorno, Chile; 3 School of Medicine, Universidad Espíritu Santo, Samborondón, Ecuador; 4 Department of Communication and Education, Universidad Loyola Andalucía, Sevilla, Spain; 5 Faculty of Education and Social Sciences, Universidad Andres Bello, Viña del Mar, Chile; 6 eFidac Grupo de de Investigación, Escuela de Educación Física, Pontificia Universidad Católica de Valparaíso, Viña del Mar, Chile; 7 Navarrabiomed, Hospital Universitario de Navarra, Universidad Pública de Navarra (UPNA), IdiSNA, Pamplona, Spain

**Keywords:** diabetes, longevity, mortality, years of potential life lost, meta-analysis

## Abstract

The aim of the study was to estimate and compare life expectancy among individuals with type 1 diabetes (T1D), type 2 diabetes (T2D) and without diabetes according to sex, geographical regions and study periods. Years of potential life lost (YPLL) was assessed as a secondary outcome. We conducted a systematic review and meta-analysis following PRISMA guidelines. PubMed, EMBASE, and Web of Science were searched from inception to June 2025 for studies reporting life expectancy in individuals with T1D, T2D, and those without diabetes. Two reviewers independently screened and extracted data. Life expectancy estimates were pooled using random-effects models with empirical Bayes estimation, and heterogeneity was quantified using the I² statistic and between-study variance (τ²). We calculated 95% confidence (CI) and prediction intervals (PI) and conducted subgroup analyses by sex and geographical region. Meta-regression assessed cohort year effects, using the median year when a range was reported, and publication bias was evaluated using Doi plots and the Luis Furuya-Kanamori (LFK) index. Twenty-three studies including 179 cohorts were identified, encompassing 65,271 individuals with T1D and over 139 million with T2D. Life expectancy was shortest among individuals with T1D: 65.12 years (95%CI: 59.51–70.72; PI: 41.22–88.98) in men and 68.26 years (95%CI: 63.01–73.51; PI: 47.26–89.23) in women. For T2D, life expectancy was 74.34 years (95%CI: 72.81–75.87; PI: 61.32–87.28) in men and 79.63 years (95%CI: 78.32–80.95; PI: 68.36–90.83) in women. By comparison, men and women without diabetes lived on average 79.56 years (95%CI: 77.98–81.15; PI: 75.81–83.46) and 84.46 years (95%CI: 83.38–85.54; PI: 81.54–87.47), respectively (all p<0.001). YPLL was 11.3 for men and 10.9 for women with T1D, and 7.0 and 6.2 for men and women with T2D, respectively. In conclusion, life expectancy remains substantially lower than in the non-diabetic population, particularly for T1D, although temporal improvements were observed. Our results highlight persistent disparities in longevity between diabetes types, sexes, and regions.

**Systematic review registration:**

https://www.crd.york.ac.uk/PROSPERO/, identifier CRD420251074407.

## 1 Introduction

Diabetes is a major global health concern, affecting an estimated 828 million individuals worldwide and contributing significantly to morbidity and mortality (1, 2), with nearly 240 million in 2021 unaware of their condition (3). Individuals with diabetes generally live shorter lives than those without, particularly those with type 1 diabetes (T1D) (4). Women, however, tend to live longer than men, both in the general population and among those with diabetes, though diabetes still significantly reduces life expectancy for both sexes (5). Multiple factors may contribute, including an increased risk of cardiovascular disease (2, 6), kidney disease (7), infections and other diabetes-related complications (8), which can be even more pronounced in specific race/ethnic groups (9). Poor glycemic control, the presence of comorbidities, and lifestyle factors such as smoking, diet, and physical activity levels also play significant roles in determining longevity (10). Over recent decades, advancements in diabetes management, including improved insulin therapies, continuous glucose monitoring, and multidisciplinary care approaches, have contributed to extended life expectancy in individuals with T1D (11) and type 2 diabetes (T2D) (12). However, disparities in healthcare access and treatment adherence continue to affect outcomes across different populations and regions (13).

The vast majority of research in this field has focused on the increased mortality risk associated with diabetes, which has been summarized in many reviews (14, 15). However, fewer studies have specifically quantified sex-related differences in life expectancy between individuals with T1D, T2D, and those without diabetes. Life expectancy is a widely used indicator of population health and offers a more intuitive understanding of the burden of diabetes by quantifying the expected number of years an individual is likely to live after diagnosis, assuming current age-specific mortality rates remain constant (16). In addition to life expectancy, the years of potential life lost (YPLL) is another important metric that highlights the years of life lost due to premature mortality. Understanding both life expectancy and YPLL in these populations is crucial for assessing the long-term impact of diabetes, guiding healthcare strategies, and informing patients and healthcare providers about prognosis (17).

To date, no meta-analysis has synthesized estimates of life expectancy and YPLL in women and men with T1D and T2D compared with those without diabetes. A clearer understanding of the impact of diabetes on longevity may inform strategies to improve care. We therefore aimed to estimate and compare life expectancy by diabetes type and sex, examining disparities across regions and study periods, and to assess YPLL as a secondary outcome.

## 2 Materials and methods

### 2.1 Protocol

This systematic review was prospectively registered in the PROSPERO (CRD420251074407) and conducted according to the PRISMA guidelines. Two investigators (YE and AG-H) independently performed the entire process from the literature selection to data extraction. Disagreements were resolved through consensus.

### 2.2 Eligibility criteria

Studies were required to meet the following PECOS criteria: (a) Participants, the population of interest included individuals diagnosed with T1D and T2D, as well as the population without diabetes serving as a comparator group. Eligible studies were required to report on individuals with a clearly defined diagnosis of diabetes and provide sex-specific estimates; (b) Exposure, diagnosis of either T1D or T2D, with no restrictions on the age of diagnosis, provided that life expectancy estimates were available; (c) The primary comparator was the general population without diabetes. However, studies comparing T1D and T2D, or comparing categories within T1D or T2D groups, were also considered, provided they included sex-specific data; (d) Outcome, the primary outcome of interest was life expectancy, either at birth or at specific ages; and (e) Study design, eligible studies included longitudinal cohort studies, registry-based studies, and large population-based studies reporting reliable life expectancy estimates, or life table models that simulate life expectancy, if they use real-world data (e.g., mortality rates, age-specific survival probabilities). Reviews, meta-analyses, case reports, small case series, studies that do not provide life expectancy data (including those that do not report estimates separately by sex) or are focused on theoretical projections without actual mortality or survival data, or studies which rely solely on stochastic modeling or theoretical simulations without any real-world data were excluded.

Searching was restricted to articles in English and Spanish languages in peer-reviewed journals.

### 2.3 Information sources, search strategy and inter-rater agreement

Two investigators (YE and AG-H) independently searched in three electronic databases (PubMed, EMBASE, and Web of Science) from inception to June 2025. The search strategy is described in detail in the **Supplementary Appendix p. 3**. Title/abstract screening and full-text assessment were done independently by two reviewers (YE and AG-H), with disagreements resolved by consensus (and a third reviewer when needed). Agreement was 88% (Cohen’s κ = 0.74) at the title/abstract stage and 93% (κ = 0.84) at full text.

### 2.4 Data collection process

Data extraction was carried out independently by both reviewers for all included studies. Each reviewer extracted all variables of interest, after which the datasets were cross-checked, and discrepancies were resolved through discussion and consensus. The following data were extracted from each study that met the selection criteria, using an Excel spreadsheet designed for the present study: (1) study characteristics (first author’s name, publication year, country, data source, study period, sample size and study design); (2) participant information (sex, age, diabetes type); and (3) methods of assessment of life expectancy. Missing data from the included studies were requested via email from the corresponding authors of the original published papers, although no replies were received.

### 2.5 Study risk of bias assessment

To evaluate risk of bias of the included studies, the Joanna Briggs Institute (JBI) appraisal checklist for cohort studies (18) was used independently by two authors. The JBI tool consists of 11 questions assessing study design, each requiring a response of ‘yes’ (indicating higher quality), ‘no’ (indicating lower quality), ‘unclear’ or ‘not applicable’. Items 4 (“Were confounding factors identified?”) and item 5 (“Were strategies to deal with confounding factors stated?”) were considered not applicable because the included studies reported aggregated life expectancy estimates rather than individual-level data, preventing the identification or adjustment for confounders. For studies that did not fully meet the traditional cohort design, other items were considered not applicable: specifically, item 9 (“Was the follow-up complete, and if not, were the reasons for loss to follow-up described and explored?”) and item 10 (“Were strategies to address incomplete follow-up utilized?”), which could not be fully assessed for studies relying solely on administrative or registry data. All other items were evaluated according to standard criteria.

### 2.6 Statistical analysis

Distributions of life expectancy are estimated and presented in forest plots. We pooled life expectancy estimates from birth to calculate the expected age of death, and when studies reported life expectancy at multiple ages, we chose the lowest available baseline age (e.g., at 40 rather than 50 or 60) to minimize potential survival bias, since older ages already exclude those who died earlier. The reported life expectancy was then added to the baseline age at which it was estimated, in order to express life expectancy as the expected age at death, rather than as years remaining. Weighted averages were estimated, and forest plots were generated using Stata version 17.0 (STATA Corp., College Station, TX). The random-effects with empirical Bayes model was used to pool the estimates of life expectancy. Subgroup analyses by geographical region were conducted only for individuals with T2D, due to the limited number of studies available for T1D. We generated forest plots of the life expectancy of individuals with T1D, T2D, and the non-diabetic population, stratified by sex. When studies reported cohort year ranges, the median year was used to standardize estimates across studies.

As a secondary outcome, we calculated YPLL whenever possible in studies that provided life expectancy estimates for both individuals with and without diabetes, determining the difference between the two groups, stratified by sex.

### 2.7 Synthesis of results

The heterogeneity index (I²) was used to estimate the percentage of variation across studies attributable to heterogeneity rather than chance. I² values of 25%, 50%, and 75% were considered low, moderate, and high degrees of heterogeneity, respectively (19). In addition, we estimated the between‐study variance (τ²) under a random‐effects model and reported 95% prediction intervals (PI) for the pooled mean to reflect the expected range of true effects in a new study. Because life expectancy is strictly positive, PIs were additionally computed on the log scale and back-transformed to years (primary presentation), ensuring non-negative limits.

### 2.8 Risk of bias across studies

Potential small-study effects arising from publication bias, substandard methodology in smaller studies, artificial associations, actual heterogeneity, or random error were evaluated using the Luis Furuya-Kanamori (LFK) test, and Doi plot. These methods have proven to be more reliable than traditional funnel plots and Egger’s regression intercept test (20). An LFK index exceeding 1 or falling below −1 signifies a minor degree of asymmetry, while values over 2 or under −2 indicate a major level of asymmetry.

## 3 Results

### 3.1 Study selection

**Figure 1:**
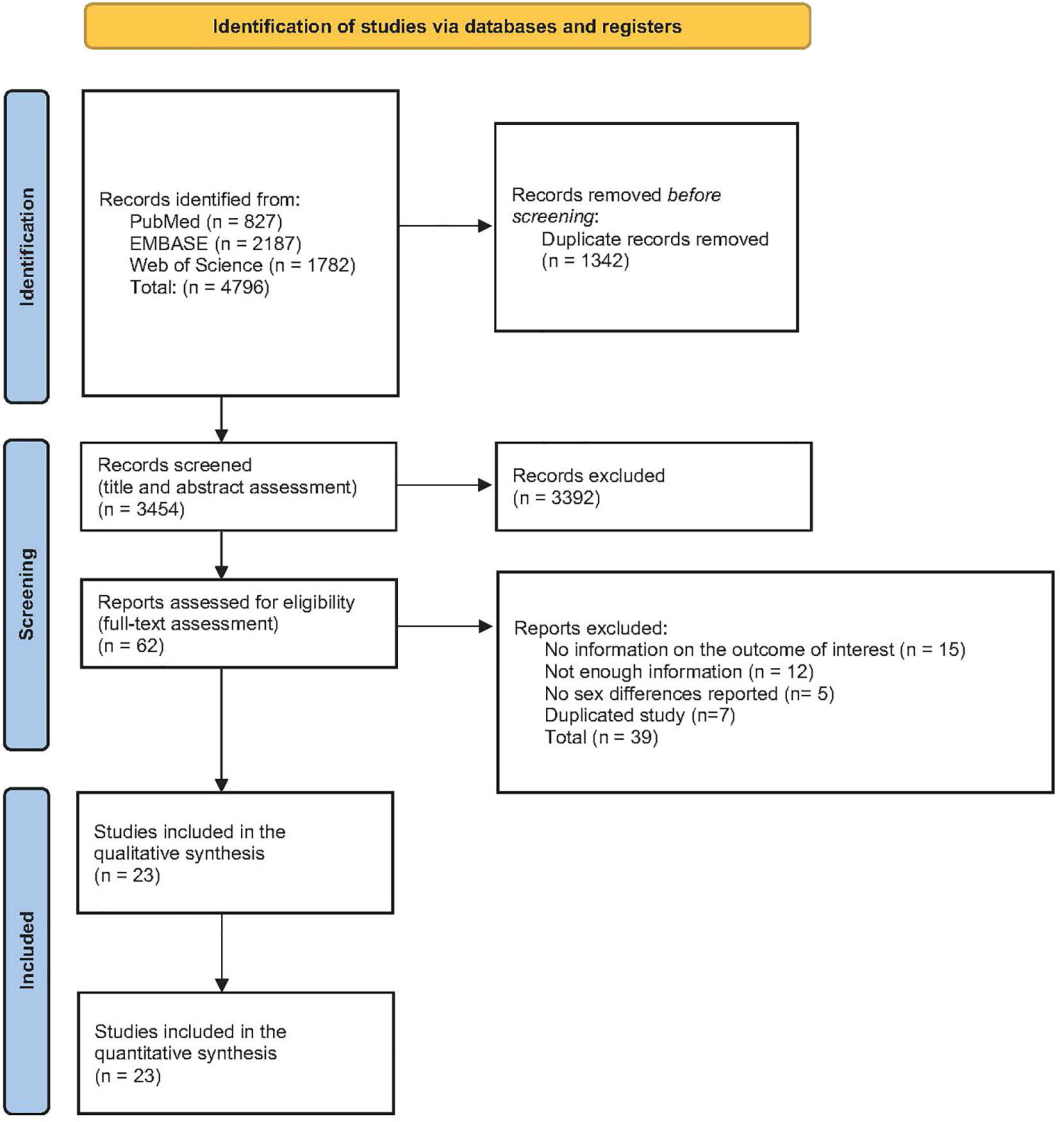
PRISMA flow diagram.

The electronic search retrieved 4,796 articles. After removing duplicates and assessing titles and abstracts, 62 studies were assessed for eligibility based on full text; and among these, 39 articles were excluded, resulting in 23 articles included in the systematic review and meta-analysis (**Figure 1**). The reference list of excluded studies and reasons for exclusion are provided in **Supplementary Appendix pp. 4-7**.

### 3.2 Study characteristics

The general characteristics of the included articles are shown in **Table 1**. In all, studies encompassed data from approximately 139,781,671 individuals with diabetes (47.9% females), and approximately 1,563,566,868 individuals without diabetes, drawn from 179 cohorts across 73 countries spanning Africa, America, Asia, Europe and Oceania. Detailed information on the specific cohorts included in each multi-country study are provided in the **Supplementary Appendix pp. 8-11**. The cohort years of the included studies ranged from 1950 to 2021, with a median study year of 2004. Population sizes varied considerably, from small longitudinal cohorts of approximately 390 to 974 participants to large national registry datasets including up to 441,199 individuals.

**Table 1:** Characteristics of included studies.

Authors (year)	Country	Data source	Study period	n with diabetes	Women with diabetes	Men with diabetes
Type 1 diabetes studies						
Brown et al. (2001) (21)	New Zealand	Canterbury Diabetes Registry’s 1984 database	1984-1993	NR	NR	NR
Huo et al. (2016) (39)	Australia	The National Diabetes Services Scheme	1997-2010	85,547	40,655	44,892
Livingstone et al. (2015) (22)	Scotland	The Scottish Care Information–Diabetes Collaboration (SCI-DC) database	2008-2010	24,691	10,765	13,926
Miller et al. (2012) EDC 1950–1964 (23)	USA	Pittsburg EDC (1950–1964)	1950–1964	390	180	210
Miller et al. (2012) EDC 1965–1980 (23)	USA	Pittsburg ACR (1965–1979)	1965–1979	543	283	260
Petrie et al. (2016) (24)	Sweden	Swedish National Diabetes Register (NDR)	2007 to 2011	27,841	11,500	14,323
Tachkov et al. (2020) (25)	Bulgaria	National Statistical Institute and National Diabetic Register from Bulgaria	2015	26,259	NR	NR
Type 2 diabetes studies						
Goto et al. (2020) (26)	Japan	Diabetes clinic	1995-2001	6,140	1,412	4,728
Hou et al. (2024) (27)	China	Henan Rural Cohort	2015-2017	1,325	844	481
Laditka et al. (2015) (28)	USA	Panel Study of Income Dynamics	1999-2001	479	311	168
Liang et al. (2020) (29)	Taiwan	Taiwan Longitudinal Study on Aging (TLSA)	1996, 1999, 2003, 2007, and 2011	559	263	296
Loukine et al. (2012) (30)	Canada	Canadian Chronic Disease Surveillance System (CCDSS) and Canadian Community Health Survey (CCHS) data.	2004-2006	NR	NR	NR
The Global Cardiovascular Risk Consortium et al. (2025) (40)	Multicountry	***(Detailed in the supplementary appendix)	1963-2021	160,079	NR	NR
Manuel et al. (2004) (31)	Canada	Ontario Diabetes Registry	1996-1997	449,211	216,658	232,553
Payne et al. (2023) (32)	South Africa	HAALSI longitudinal cohort	2015-2018	557	326	231
Preston et al. (2018) (33)	USA	National Health interview Survey and Linked Mortality Files (NHIS-LMF)	1997-2009	146,559	61,090	85,469
Price et al. (2010) (34)	UK	UK Lipids in Diabetes Study	1999-2001	4,026	NR	NR
Tachkov et al. (2020) (25)	Bulgaria	National Statistical Institute and National Diabetic Register from Bulgaria	2015	441,199	261,361	206,097
Tian et al. (2024) (35)	China	The China Chronic Disease and Risk Factors Surveillance	2013-2014	14,217	10,456	3,761
Tomic et al. (2022) (41)	Multicountry	***(Detailed in the supplementary appendix)	2005-2019	138,088,545	NR	NR
Turin et al. (2012) (36)	Japan	Nippon data80 (National Integrated Project for Prospective Observation of Non-communicable Disease And its Trends in the Aged)	1980	797	278	519
Walker et al. (2018) (42)	Scotland	Scottish Care Information Diabetes database	2012-2014	272,597	306,872	390,567
Wright et al. (2017) (37)	UK	The Clinical Practice Research Datalink	1998-2015	187,968	84,228	103,740
Wubishet et al. (2021) (38)	Australia	Australian Longitudinal Study on Women’s Health (ALSWH)	1996, 1999, 2002, 2005, 2008, 2011 until 2019	974	974	0

*** Detailed in the Supplementary Appendix.

With respect to types of diabetes, seven cohorts analyzing life expectancy in individuals with T1D (21–25, 39), with data of 165,271 patients, were included. These studies were conducted in New Zealand (21), Australia (39), Scotland (22), USA (23), Sweden (24) and Bulgaria (25).

Additionally, there were 18 studies analyzing life expectancy in individuals with T2D (25–38, 40–42), several of which encompassed large-scale population datasets or multiple cohorts, yielding data from 139,616,400 participants. These studies were conducted in USA (28, 33), Japan (26, 36), China (27, 35), Australia (38), UK (34, 37), Taiwan (29), Canada (30, 31), South Africa (32), Bulgaria (25) Scotland (42), and multi-countries (40, 41).

### 3.3 Summary measures

**Figure 2:**
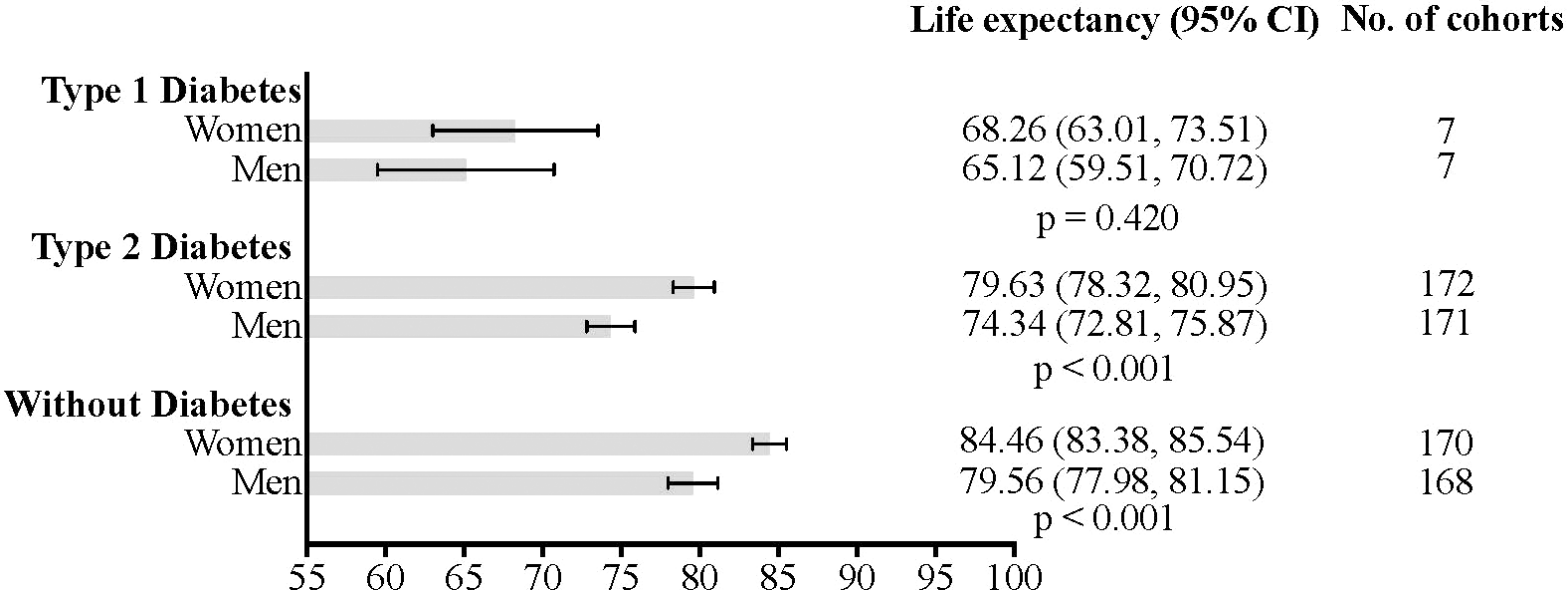
Estimated life expectancy and 95% confidence intervals for individuals with type 1 diabetes, type 2 diabetes, and without diabetes.

The primary summary measure was life expectancy for individuals with T1D or T2D, and for those without diabetes (**Figure 2**). Life expectancy was obtained by adding the reported life expectancy to the baseline age at which it was estimated in each study, and results are presented with their associated 95% CI. YPLL were additionally calculated as the difference in life expectancy between individuals without diabetes and those with T1D or T2D.

### 3.4 Risk of bias within studies

Each study fulfilled a minimum of seven out of nine applicable criteria on the JBI tool, reflecting good methodological quality. The average score across studies was 9 out of 11 (**Supplementary Appendix pp. 12-13**).

### 3.5 Synthesis of results

As shown in **Supplementary Appendix p.14**, individuals with T1D had the shortest life expectancy, with men living an average of 65.12 years (95%CI: 59.51–70.72; I^2^ = 99.78; τ²=75.25; PI = 41.22 to 88.98) and women 68.26 years (95%CI: 63.01–73.51; I^2^ = 99.70; τ²=58.11; PI = 47.26 to 89.23) (p=0.420 for the difference).

In T2D, men had an estimated life expectancy of 74.34 years (95%CI: 72.81–75.87; I^2^ = 100.00; τ²=39.15; PI = 61.32 to 87.28), while women lived 79.63 years (95%CI: 78.32–80.95; I^2^ = 100.00; τ²=29.44; PI = 68.36 to 90.83) (p < 0.001 for the difference) (**Supplementary Appendix p.15**).

As expected, individuals without diabetes had the highest life expectancy, with women living significantly longer than men (p<0.001): 84.46 years (95%CI: 83.38–85.54; I^2^ = 99.99; τ²=2.02; PI = 81.54 to 87.47) compared to 79.56 years (95%CI: 77.98–81.15; I^2^ = 99.96; τ²=3.36; PI = 75.81 to 83.46) (**Supplementary Appendix p. 16**). Overall, these findings indicate that life expectancy is lowest in both men and women with T1D, followed by those with T2D, while individuals without diabetes have the highest life expectancy (all p-values <0.001) (**Supplementary Appendix pp. 17-22**).

Subgroup analysis according to geographical region showed that among women with T2D, the highest life expectancy was observed in Asia (81.40 years [95%CI: 79.67-83.13]) (I^2^ = 100.00; τ²=38.71; PI = 66.35 to 96.46), followed by Europe (78.83 years [95%CI: 76.87-80.79]) (I^2^ = 99.87; τ²=13.07; PI = 70.57 to 87.09), and North America (78.26 years [95%CI: 74.29-82.23]) (I^2^ = 99.85; τ²=2.26; PI = 71.42 to 85.03) (**Supplementary Appendix p.23**). Among men with T2D, life expectancy was 75.72 years (95%CI: 73.48-77.95) (I^2^ = 100.00; τ²=54.80; PI = 57.72 to 93.55) in Asia and 74.11 years (95%CI: 71.89-76.33) (I^2^ = 99.85; τ²=13.17; PI = 65.82 to 82.40) in Europe (**Supplementary Appendix p. 24**). There were not enough studies with data from T1D to conduct analyses according to geographical region. Regarding individuals without diabetes, men from Asia had the highest life expectancy (82.36 years [95%CI: 80.18-84.55]) (I^2^ = 99.87; τ²=12.87; PI = 73.05 to 91.68), and the lowest was observed in Africa (71.78 years [95%CI: 70.17-73.39]) (I^2^ = 0; τ²=0; PI = 70.17 to 73.39) (**Supplementary Appendix p. 25**). In women without diabetes, the highest life expectancy was observed in Asia (86.96 years [95%CI: 85.28-88.65]) (I^2^ = 99.80; τ²=7.56; PI = 79.82 to 94.09), and the lowest in Africa (78.69 years [95%CI: 77.37-80.01]) (I^2^ = 0; τ²=0; PI = 77.37 to 80.01) (**Supplementary Appendix p. 26**).

**Figure 3:**
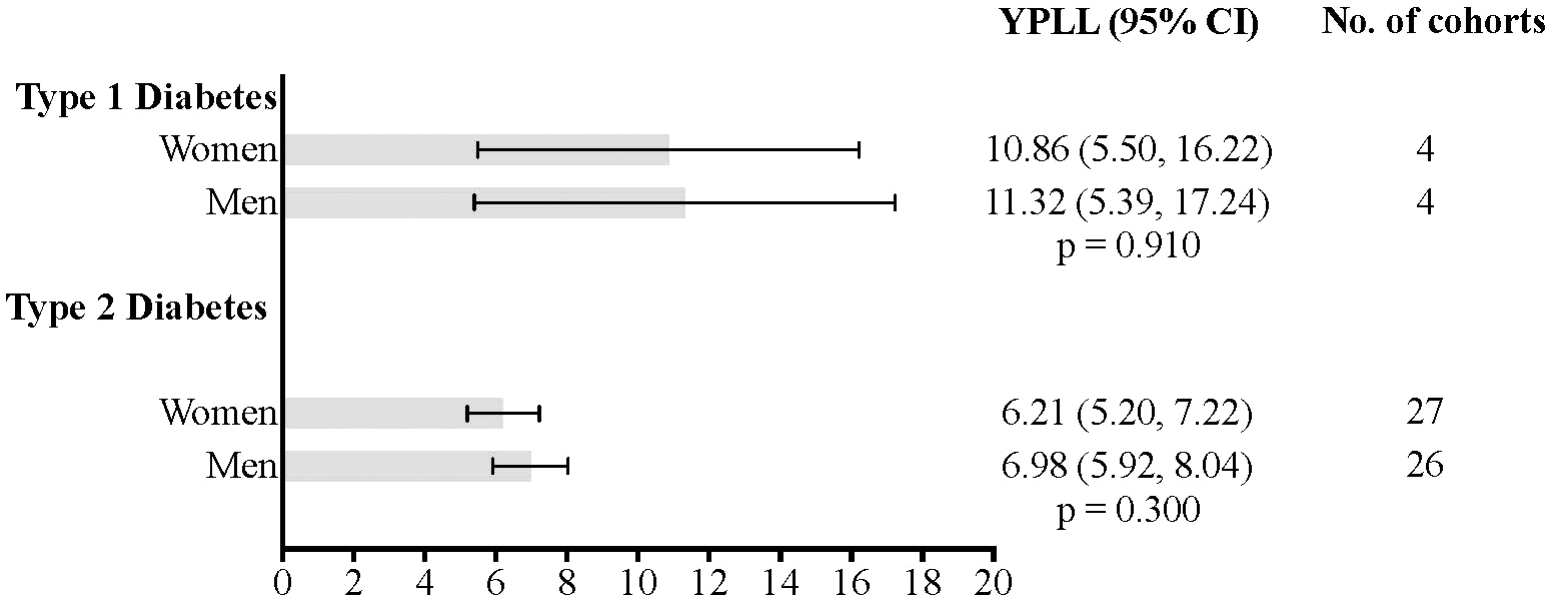
Estimated years of potential life lost and 95% confidence intervals for individuals with type 1 diabetes, type 2 diabetes.

Sex-specific estimates of YPLL for individuals with T1D and T2D are presented in **Supplementary Appendix pp. 27, 28**, respectively (**Figure 3**). Among those with T1D, men had a YPLL of 11.32 (95%CI: 5.39-17.24) (I^2^ = 99.44; τ²=0.07; PI = 5.61 to 18.54), while women had a YPLL of 10.86 (95%CI: 5.50-16.22) (I^2^ = 99.72; τ²=0.22; PI = 3.40 to 27.67) (p=0.910 for the difference). For T2D, men experienced 6.98 YPLL (95%CI: 5.92-8.04) (I^2^ = 99.99; τ²=3.88; PI = 2.81 to 11.10), whereas women had 6.21 YPLL (95%CI: 5.20-7.22) (I^2^ = 99.99; τ²=1.00; PI = 4.11 to 8.33) (p=0.300 for the difference).

### 3.6 Meta-regression analysis and publication bias

A random-effects meta-regression was conducted to assess the influence of cohort year on life expectancy across individuals with T1D and T2D, and also in individuals without diabetes, as shown in **Supplementary Appendix pp. 29-31**. In women with T1D, the results revealed a statistically significant positive association between cohort year and life expectancy (beta=0.272, p=0.001), indicating that life expectancy increased by 0.272 years for each additional cohort year. In men with T1D, a similar significant positive relationship was found (beta=0.286, p=0.002). Conversely, no significant association between cohort year and life expectancy was found for individuals with T2D, whether in women (beta=0.019, p=0.851) or men (beta=0.018, p=0.856). Similarly, no association was observed among individuals without diabetes, either in women (beta=0.019, p=0.765) or in men (beta=0.052, p=0.574).

Substantial asymmetry indicative of small-study effects was observed in the life expectancy estimates for individuals with T1D (LFK = 8.36) and for those without diabetes (LFK=–2.67). In contrast, studies involving individuals with T2D showed only minor asymmetry (LFK=–1.56) (**Supplementary Appendix pp. 20–21**).

## 4 Discussion

Our meta-analysis provides estimates of life expectancy and YPLL as secondary outcome in individuals with T1D and T2D, as well as in the population without diabetes, stratified by sex. The findings highlight significant disparities in life expectancy and YPLL, with T1D showing the most substantial reduction compared to individuals without diabetes, followed by T2D, which presents a comparatively smaller decrease. Discrepancies in life expectancy among individuals with T2D are observed across geographical regions, and findings suggest that advances in the treatment of T1D have led to a significant year-on-year increase in life expectancy.

Individuals with T1D face a significantly greater reduction in life expectancy and YPLL compared to both the general population and those with T2D. The larger reduction in life expectancy and YPLL among individuals with T1D reflects key differences in disease pathophysiology. T1D results from absolute insulin deficiency, requiring lifelong exogenous insulin therapy (43). The prolonged exposure to hyperglycemia in T1D contributes to a higher burden of complications, including cardiovascular disease and severe hypoglycemia (44), with cardiovascular disease and renal disease being the leading causes of mortality (2, 45). In contrast, T2D, characterized by insulin resistance and progressive beta-cell dysfunction (46), typically developing later in life and influenced by lifestyle factors (47), is associated with a comparatively smaller reduction in YPLL, estimated at around six years. While chronic complications such as cardiovascular disease and renal disease remain significant (48, 49), their impact is less severe compared to T1D. Much of the improvement in life expectancy over the past decades can be attributed to advances in diabetes treatment, including intensive insulin therapy, better glucose monitoring, and management of cardiovascular risk factors. These historical changes have substantially reduced excess mortality, particularly from cardiovascular disease, in high-income countries. However, under current diabetes care, the focus has shifted to managing emerging causes of death, such as infections, cancer, liver disease, and dementia, which now contribute more prominently to mortality among people with diabetes (50, 51). However, with even modest lifestyle changes after diagnosis (52), including glycemic control, proper management of comorbidities, and weight loss, many individuals with T2D can achieve a near-normal lifespan (53). Accordingly, life expectancy can vary substantially between individuals, and patients who maintain strict glycemic control and actively manage comorbidities often experience more favorable outcomes than the population averages suggest.

Sex differences in life expectancy are evident across all groups, with women generally outliving men; however, diabetes narrows this gap. Among individuals with T1D, women were found to live approximately three years longer than men and showing similar years of life lost (10.86 vs 11.32 years). This finding suggests that T1D attenuates the typical female survival advantage observed in the general population. This attenuation likely reflects a combination of biological, clinical, and behavioral factors, as chronic hyperglycemia and insulin deficiency may accelerate ovarian aging and lead to earlier menopause (54, 55), reducing the duration of estrogen-related cardioprotection (56). This contrasts with T2D, where women tend to retain this protective effect longer (57), contributing to a statistically significant greater difference in life expectancy between men and women, approximately six years. This difference may also result from a combination of behavioral, and psychological factors. Women often engage more actively with healthcare services, adhere better to treatment regimens, and adopt healthier lifestyle habits, which may mitigate the impact of diabetes on longevity (58). They may also employ more effective coping strategies and rely on social networks, buffering the effects of emotional stress (59). However, although overall sex differences in life expectancy were small and non-significant in our analysis, previous literature has reported higher cause-specific mortality risks in women with T1D suggesting that future studies should assess sex disparities at the cause-specific level.

The analysis by geographical region highlights significant variability in life expectancy among individuals with T2D. Women consistently outlive men across all regions, with the longest life expectancy observed in Oceania and the shortest in Europe. Notably, women with T2D in Oceania and Asia tend to live longer than their counterparts in Europe and North America, while among men, life expectancy is highest in Asia and lowest in Europe. These regional differences may reflect variations in cultural and lifestyle factors (60), genetic predispositions (61), and socioeconomic conditions (42). Individuals with lower education or socioeconomic status are at higher risk of developing T2D and often face a greater disease burden, creating a “vicious cycle” of disadvantage (62). Differences in healthcare access and resources between high- and low-income countries further contribute to these patterns, as fewer than one in ten people with diabetes in low- and middle-income countries receive guideline-based treatment (63). Disparities in healthcare systems and access to medications, particularly in insurance-financed systems, can exacerbate risks, further impacting life expectancy in these patients (64).

To examine variations in life expectancy across study periods, we conducted meta-regressions, as decade-specific estimates were not feasible due to the limited number of studies in some periods. The positive association observed between later cohort year and higher life expectancy in individuals with T1D, is likely attributable to advancements in the management and treatment of diabetes. Earlier cohorts, particularly those dating back to the 1960s and 1970s, reflect periods when life expectancy was generally lower due to limited therapeutic options, less structured diabetes care, and the absence of modern technologies. In contrast, more recent decades have seen major advancements, including blood glucose meters, insulin pumps, continuous glucose monitors, and more recently, systems that combine both a pump and a monitor for algorithm-driven automation of insulin delivery (65, 66). In parallel, there has been substantial progress in the prevention, early detection, and treatment of diabetes-related complications and comorbidities, such as cardiovascular disease, kidney disease, and retinopathy, which has also contributed to improved survival. However, the incidence of T1D is rising globally (66), potentially due to environmental factors such as obesity, cholesterol levels, early-life exposures, and environmental pollutants. Therefore, despite improvements, the ongoing gap in life expectancy highlights the need for continued innovations in diabetes care and prevention strategies. However, cohort year and life expectancy in individuals with T2D were not significantly associated, potentially due to several factors. First, T2D is often diagnosed later in life, leaving less time for treatments to impact life expectancy (67). Additionally, while newer treatments are available [e.g., SGLT2 inhibitors and GLP-1 receptor agonists (68)], their widespread adoption is still recent, which may explain the lack of noticeable improvements in life expectancy over time. Finally, the presence of other health conditions, such as obesity and hypertension, may counteract the benefits of diabetes management, limiting the positive effects on life expectancy (69).

### 4.1 Limitations

Our meta-analysis has several strengths, including its large sample size of over one hundred million participants, which enhances generalizability and statistical power. However, some limitations should be noted. First, the number of studies for T1D is modest (k = 6), and some regional T2D strata include few cohorts. This small k reduces power, widens confidence and prediction intervals, and limits assessment of heterogeneity and small-study bias. Second, most studies relied on administrative data, lacking key clinical risk factors (e.g., glycated hemoglobin, lipid profile, renal function, BMI, smoking status, or diabetes-related complications) that could influence survival estimates. Third, although data from countries with universal healthcare systems may reduce selection bias, our findings may not generalize to settings with different healthcare models, such as the United States, where disparities in diabetes care and treatment affordability could affect outcomes. Fourth, the inclusion of very old cohorts, whose outcomes were shaped by markedly different standards of care compared to current practice, may contribute to heterogeneity, and limit the direct comparability of results across studies. In addition, life expectancy was standardized to birth across studies, which may introduce some survivor bias, particularly for T2D, as life expectancy from birth includes years before typical disease onset. Most studies did not separate pre-onset years, but this is unlikely to substantially affect overall patterns, since mortality differences are largely driven by mid- and late-life deaths. Fifth, as all stages of screening and data extraction were performed by only two reviewers, the possibility of reviewer bias cannot be entirely excluded, although independent extraction and cross-checking were used to minimize this risk. Sixth, life expectancy estimates for T1D may not apply globally due to scarce and outdated data from low- and middle-income countries, as highlighted by The Lancet Commission (70). Furthermore, differences in study methodologies should also be considered when interpreting our findings. Life expectancy was estimated using varying approaches, and definitions of T1D were not uniform, with some relying on age at diagnosis or registry codes, which may have led to the inclusion of individuals with late-onset autoimmune diabetes or misclassified T2D. Similarly, variability in data source, ranging from nationwide registries to small cohorts, may influence the representativeness of the study populations. Additionally, undiagnosed diabetes (3) could bias estimates for the non-diabetic comparison group. Sixth, in several included studies, the number of deaths and person-time at age extremes was likely limited. However, most studies did not provide detailed information on how such sparse data were handled, including whether imputation, smoothing, or extrapolation techniques were applied. This lack of transparency introduces uncertainty, as the treatment of limited data at these age ranges can substantially influence life expectancy estimates. Additionally, our study does not differentiate between individuals with well-controlled versus poorly controlled diabetes, and therefore may not reflect the higher life expectancy achievable in patients maintaining strict glycemic control. Finally, YPLL could not be calculated in all studies due to the lack of sex-stratified life expectancy data. Finally, while adults with diabetes had shorter life expectancy than those without, evidence suggests that optimal control of multiple modifiable risk factors can substantially reduce excess mortality, particularly in T2D (40).

## 5 Conclusion

Our findings confirm that diabetes significantly reduces life expectancy, with T1D associated with the greatest reduction, followed by T2D. The analysis of YPLL further underscores these disparities, revealing substantial years of potential life lost in individuals with diabetes. Although women generally live longer than men, the sex-based longevity advantage is diminished in those with diabetes. These findings highlight the urgent need for targeted interventions to increase lifespan and reduce premature mortality in this population. For T1D, efforts should focus on optimizing glycemic control, improving cardiovascular risk management, and addressing care disparities. For T2D, emphasis should be on early diagnosis, lifestyle changes, and implementing cardioprotective therapies. Collaboration between policymakers and healthcare providers is essential to ensure equitable access to diabetes care, especially for high-risk populations. Future research on new treatments, lifestyle interventions, and emerging technologies like artificial intelligence, telemedicine or artificial pancreas systems are crucial to improving life expectancy in people with diabetes.

## Data Availability

The original contributions presented in the study are included in the article/**Supplementary Material**. Further inquiries can be directed to the corresponding author.
