# Supplementary material for: Life expectancy in individuals with type 1, type 2 diabetes and without diabetes: a systematic review and meta-analysis
Source: Front Endocrinol (Lausanne). 2025 Nov 13;16:1704277. doi: 10.3389/fendo.2025.1704277 (PMC12658360; doi:10.3389/fendo.2025.1704277)
Supplement: Supplementary file 1 [file DataSheet1.docx]

**ELECTRONIC SUPPLEMENTARY MATERIAL**

**Life expectancy in individuals with type 1 and 2 diabetes and without diabetes: Systematic Review and Meta-Analysis.**

Contents

[Supplementary Table 1. Literature search strategy for all the databases 3](#_Toc211342866)

[Supplementary Table 2. Excluded studies and reason for exclusion. 4](#_Toc211342867)

[Supplementary Table 3. Cohort characteristics by multi-country studies. 8](#_Toc211342868)

[Supplementary Table 4. Results of the Joanna Briggs Institute (JBI) Appraisal Checklist for Cohort studies. 12](#_Toc211342869)

[Supplementary Figure 1. Forest plot showing life expectancy estimates and its 95% confidence interval of individuals with type 1 diabetes according to sex. 14](#_Toc211342870)

[Supplementary Figure 2. Forest plot showing life expectancy estimates and its 95% confidence interval of individuals with type 2 diabetes according to sex. 15](#_Toc211342871)

[Supplementary Figure 3. Forest plot showing life expectancy estimates and its 95% confidence interval of individuals without diabetes according to sex. 16](#_Toc211342872)

[Supplementary Figure 4. Forest plot showing the comparison between life expectancy estimates and 95% confidence interval of men with type 1 diabetes and type 2 diabetes. 17](#_Toc211342873)

[Supplementary Figure 5. Forest plot showing the comparison between life expectancy estimates and 95% confidence interval of men with type 1 diabetes and without diabetes. 18](#_Toc211342874)

[Supplementary Figure 6. Forest plot showing the comparison between life expectancy estimates and 95% confidence interval of men with type 2 diabetes and without diabetes. 19](#_Toc211342875)

[Supplementary Figure 7. Forest plot showing the comparison between life expectancy estimates and 95% confidence interval of women with type 1 diabetes and type 2 diabetes. 20](#_Toc211342876)

[Supplementary Figure 8. Forest plot showing the comparison between life expectancy estimates and 95% confidence interval of women with type 1 diabetes and without diabetes. 21](#_Toc211342877)

[Supplementary Figure 9. Forest plot showing the comparison between life expectancy estimates and 95% confidence interval of women with type 2 diabetes and without diabetes. 22](#_Toc211342878)

[Supplementary Figure 10. Forest plot showing life expectancy estimates and its 95% confidence interval of women with type 2 diabetes according to geographical area. 23](#_Toc211342879)

[Supplementary Figure 11. Forest plot showing life expectancy estimates and its 95% confidence interval of men with type 2 diabetes according to geographical area. 24](#_Toc211342880)

[Supplementary Figure 12. Forest plot showing life expectancy estimates and its 95% confidence interval of men without diabetes according to geographical area. 25](#_Toc211342881)

[Supplementary Figure 13. Forest plot showing life expectancy estimates and its 95% confidence interval of women without diabetes according to geographical area. 26](#_Toc211342882)

[Supplementary Figure 14. Forest plot showing the years of potential life lost estimates and 95% confidence interval of individuals with type 1 diabetes. 27](#_Toc211342883)

[Supplementary Figure 15. Forest plot showing the years of potential life lost estimates and 95% confidence interval of individuals with type 2 diabetes. 28](#_Toc211342884)

[Supplementary Figure 16. Meta-regression analysis of a) cohort period and life expectancy in men with type 1 diabetes; b) cohort period and life expectancy in women with type 1 diabetes; c) cohort period and life expectancy in men with type 2 diabetes; d) cohort period and life expectancy in women with type 2 diabetes; e) cohort period and life expectancy in men without diabetes; and f) cohort period and life expectancy in women without diabetes. The solid line indicates a linear relationship. The size of each data point is proportional to its statistical weight. 29](#_Toc211342885)

[Supplementary Figure 17. LFK Index and Doi plots for publication bias detection in a) Type 1 diabetes individuals; b) Type 2 diabetes individuals; and c) Diabetes-free population. 32](#_Toc211342886)

This supplemental material has been provided by the authors to give readers additional information about their work.

# **Supplementary Table 1.** Literature search strategy for all the databases

| PubMed:  #1: Diabetes Mellitus [MeSH] OR "type 2 diabetes" OR “type 1 diabetes” OR “T1DM” OR "T2DM"  #2: "life expectancy" OR " longevity"  #3: "prospective" OR "retrospective" OR "cohort" OR "case-control"  (#1 AND #2 AND #3) | 827 results |
| --- | --- |
| Embase:  #1 'diabetes mellitus, type 2'/exp OR 'type 2 diabetes' OR 't2dm' OR 'type 1 diabetes' OR 't1dm' OR 'diabetes mellitus'  #2 “life expectancy" OR " longevity"  #3 'cohort analysis'/de OR 'cross sectional study'/de OR 'longitudinal study'/de OR 'observational study'/de OR 'prospective study'/de OR 'retrospective study'/de  (#1 AND #2 AND #3) | 2187 results |
| Web of Science:  ("type 2 diabetes" OR "T2DM" OR "diabetes mellitus" OR “type 1 diabetes” OR “T1DM”) AND (“life expectancy") AND ("prospective study" OR "retrospective study" OR "cohort study" OR "case-control study" OR prospective OR retrospective OR cohort OR "case-control") | 1782 results |

# **Supplementary Table 2.** Excluded studies and reason for exclusion.

| Nº | Study | Reason for exclusion |
| --- | --- | --- |
| 1 | Alam, M. Z., & Sheoti, I. H. (2024). The burden of diabetes and hypertension on healthy life expectancy in Bangladesh. Scientific Reports, 14(1), 7936. | No outcome of interest |
| 2 | Andrade, F. (2009). Estimating diabetes and diabetes-free life expectancy in Mexico and seven major cities in Latin America and the Caribbean. Revista Panamericana de Salud Pública, 26, 9-16. | No sex differences |
| 3 | Andrade, F. C. (2010). Measuring the impact of diabetes on life expectancy and disability-free life expectancy among older adults in Mexico. Journals of Gerontology Series B: Psychological Sciences and Social Sciences, 65(3), 381-389. | Duplicated study |
| 4 | Arffman, M., Hakkarainen, P., Keskimäki, I., Oksanen, T., & Sund, R. (2023). Long-term and recent trends in survival and life expectancy for people with type 1 diabetes in Finland. Diabetes Research and Clinical Practice, 198, 110580. | No sex differences reported |
| 5 | Asllanaj, E., Bano, A., Glisic, M., Jaspers, L., Ikram, M. A., Laven, J. S., ... & Franco, O. H. (2019). Age at natural menopause and life expectancy with and without type 2 diabetes. Menopause, 26(4), 387-394. | Not enough information |
| 6 | Canudas-Romo, V., García-Guerrero, V. M., & Echarri-Cánovas, C. J. (2015). The stagnation of the Mexican male life expectancy in the first decade of the 21st century: the impact of homicides and diabetes mellitus. J Epidemiol Community Health, 69(1), 28-34. | No outcome of interest |
| 7 | Díaz-Venegas, C., Schneider, D. C., Myrskylä, M., & Mehta, N. K. (2017). Life expectancy with and without cognitive impairment by diabetes status among older Americans. PLoS One, 12(12), e0190488. | Not enough information |
| 8 | Franco, O. H., Steyerberg, E. W., Hu, F. B., Mackenbach, J., & Nusselder, W. (2007). Associations of diabetes mellitus with total life expectancy and life expectancy with and without cardiovascular disease. Archives of internal medicine, 167(11), 1145-1151. | Duplicated study |
| 9 | Harding, J. L., Shaw, J. E., Peeters, A., Guiver, T., Davidson, S., & Magliano, D. J. (2014). Mortality trends among people with type 1 and type 2 diabetes in Australia: 1997–2010. Diabetes care, 37(9), 2579-2586. | No outcome of interest |
| 10 | Hayes, A. J., Leal, J., Kelman, C. W., & Clarke, P. M. (2011). Risk equations to predict life expectancy of people with type 2 diabetes mellitus following major complications: a study from Western Australia. Diabetic medicine, 28(4), 428-435. | No outcome of interest |
| 11 | Höhn, A., McGurnaghan, S. J., Caparrotta, T. M., Jeyam, A., O’Reilly, J. E., Blackbourn, L. A., ... & SDRN-Epi Group. (2022). Large socioeconomic gap in period life expectancy and life years spent with complications of diabetes in the Scottish population with type 1 diabetes, 2013–2018. PLoS One, 17(8), e0271110. | Duplicated study |
| 12 | Huo, L., Shaw, J. E., Wong, E., Harding, J. L., Peeters, A., & Magliano, D. J. (2016). Burden of diabetes in Australia: life expectancy and disability-free life expectancy in adults with diabetes. Diabetologia, 59, 1437-1445. | Duplicated study |
| 13 | Ioacara, S., Lichiardopol, R., Ionescu-Tirgoviste, C., Cheta, D., Sabau, S., Guja, C., ... & Tiu, C. (2009). Improvements in life expectancy in type 1 diabetes patients in the last six decades. diabetes research and clinical practice, 86(2), 146-151. | Not enough information |
| 14 | Ioacara, S., Guja, C., Ionescu-Tirgoviste, C., Fica, S., Sabau, S., Radu, S., ... & Tiu, C. (2011). Improvements in life expectancy in adult type 2 diabetes patients in the last six decades. diabetes research and clinical practice, 92(3), 400-404. | Not enough information |
| 15 | Ioacara, S., Guja, C., Fica, S., & Ionescu-Tirgoviste, C. (2013). The dynamics of life expectancy over the last six decades in elderly people with diabetes. Diabetes Research and Clinical Practice, 99(2), 217-222. | Not enough information |
| 16 | Jagger, C., Goyder, E., Clarke, M., Brouard, N., & Arthur, A. (2003). Active life expectancy in people with and without diabetes. Journal of public health, 25(1), 42-46. | No sex differences reported |
| 17 | Jia, H., Zack, M. M., & Thompson, W. W. (2013). The effects of diabetes, hypertension, asthma, heart disease, and stroke on quality-adjusted life expectancy. Value in health, 16(1), 140-147. | No outcome of interest |
| 18 | Jonker, J. T., De Laet, C., Franco, O. H., Peeters, A., Mackenbach, J., & Nusselder, W. J. (2006). Physical activity and life expectancy with and without diabetes: life table analysis of the Framingham Heart Study. Diabetes care, 29(1), 38-43. | Duplicated study |
| 19 | Kang, Y. M., Cho, Y. K., Lee, S. E., Park, J. Y., Lee, W. J., Kim, Y. J., & Jung, C. H. (2017). Cardiovascular diseases and life expectancy in adults with type 2 diabetes: a Korean national sample cohort study. The Journal of Clinical Endocrinology & Metabolism, 102(9), 3443-3451. | No outcome of interest |
| 20 | Leal, J., Gray, A. M., & Clarke, P. M. (2009). Development of life-expectancy tables for people with type 2 diabetes. European heart journal, 30(7), 834-839. | No outcome of interest |
| 21 | Lutgers, H. L., Gerrits, E. G., Sluiter, W. J., Ubink-Veltmaat, L. J., Landman, G. W., Links, T. P., ... & Bilo, H. J. (2009). Life expectancy in a large cohort of type 2 diabetes patients treated in primary care (ZODIAC-10). PLoS One, 4(8), e6817. | Not enough information |
| 22 | Manuel, D. G., & Schultz, S. E. (2004). Health-related quality of life and health-adjusted life expectancy of people with diabetes in Ontario, Canada, 1996–1997. Diabetes care, 27(2), 407-414. | No outcome of interest |
| 23 | Muschik, D., Tetzlaff, J., Lange, K., Epping, J., Eberhard, S., & Geyer, S. (2017). Change in life expectancy with type 2 diabetes: a study using claims data from lower Saxony, Germany. Population Health Metrics, 15, 1-10. | Not enough information |
| 24 | Ou, H. T., Yang, C. Y., Wang, J. D., Hwang, J. S., & Wu, J. S. (2016). Life expectancy and lifetime health care expenditures for type 1 diabetes: a nationwide longitudinal cohort of incident cases followed for 14 years. Value in Health, 19(8), 976-984. | Not enough information |
| 25 | Perna, L., Thien-Seitz, U., Ladwig, K. H., Meisinger, C., & Mielck, A. (2010). Socio-economic differences in life expectancy among persons with diabetes mellitus or myocardial infarction: results from the German MONICA/KORA study. BMC public health, 10, 1-11. | Duplicated study |
| 26 | Rapoport, M., Chetrit, A., Cantrell, D., Novikov, I., Roth, J., & Dankner, R. (2021). Years of potential life lost in pre-diabetes and diabetes mellitus: data from a 40-year follow-up of the Israel study on glucose intolerance, obesity and hypertension. BMJ Open Diabetes Research and Care, 9(1), e001981. | Not enough information |
| 27 | Salvador Jr, D., Bano, A., Wehrli, F., Gonzalez-Jaramillo, V., Laimer, M., Hunziker, L., & Muka, T. (2023). Impact of type 2 diabetes on life expectancy and role of kidney disease among inpatients with heart failure in Switzerland: an ambispective cohort study. Cardiovascular diabetology, 22(1), 174. | No outcome of interest |
| 28 | Sorbi, M. H., Rahmanian, M., Sadeghi, K., Ahmadi, S. M., Baghaeipour, L., & Yazdanpoor, S. (2014). Comparison of the life expectancy and general health in type 2 diabetic patients with non-patients. Iranian Journal of Diabetes and Obesity, 6(3), 114-118. | Not enough information |
| 29 | Sussman, J. B., Kerr, E. A., Saini, S. D., Holleman, R. G., Klamerus, M. L., Min, L. C., ... & Hofer, T. P. (2015). Rates of deintensification of blood pressure and glycemic medication treatment based on levels of control and life expectancy in older patients with diabetes mellitus. JAMA internal medicine, 175(12), 1942-1949. | No outcome of interest |
| 30 | Tönnies, T., Baumert, J., Heidemann, C., von der Lippe, E., Brinks, R., & Hoyer, A. (2021). Diabetes free life expectancy and years of life lost associated with type 2 diabetes: projected trends in Germany between 2015 and 2040. Population health metrics, 19(1), 38. | No outcome of interest |
| 31 | Tran-Duy, A., Knight, J., Clarke, P. M., Svensson, A. M., Eliasson, B., & Palmer, A. J. (2021). Development of a life expectancy table for individuals with type 1 diabetes. Diabetologia, 64, 2228-2236. | No outcome of interest |
| 32 | Tsukinoki, R., Murakami, Y., Hayakawa, T., Kadota, A., Harada, A., Kita, Y., ... & Ueshima, H. (2025). Comprehensive assessment of the impact of blood pressure, body mass index, smoking, and diabetes on healthy life expectancy in Japan: NIPPON DATA90. Journal of Epidemiology, JE20240298. | Duplicated study |
| 33 | Wan, E. Y. F., Chin, W. Y., Yu, E. Y. T., Wong, I. C. K., Chan, E. W. Y., Li, S. X., ... & Lam, C. L. K. (2020). The impact of cardiovascular disease and chronic kidney disease on life expectancy and direct medical cost in a 10-year diabetes cohort study. Diabetes Care, 43(8), 1750-1758. | No outcome of interest |
| 34 | Wang, B., Fu, Y., Tan, X., Wang, N., Qi, L., & Lu, Y. (2024). Assessing the impact of type 2 diabetes on mortality and life expectancy according to the number of risk factor targets achieved: an observational study. BMC medicine, 22(1), 114. | No outcome of interest |
| 35 | Wen, C. P., Chang, C. H., Tsai, M. K., Lee, J. H., Lu, P. J., Tsai, S. P., ... & Wu, X. (2017). Diabetes with early kidney involvement may shorten life expectancy by 16 years. Kidney international, 92(2), 388-396. | Not enough information |
| 36 | You, W. P., & Henneberg, M. (2016). Type 1 diabetes prevalence increasing globally and regionally: the role of natural selection and life expectancy at birth. BMJ open diabetes research and care, 4(1), e000161. | Not enough information |
| 37 | Yu, D., Zhao, Z., Pickering, K., Baker, J., Cutfield, R., Orr-Walker, B. J., ... & Simmons, D. (2024). Association between within-target risk factors and life expectancy free from cardiovascular disease, cancer, and dementia in individuals with type 2 diabetes in New Zealand between 1994 and 2018: a multi-ethnic cohort study. BMC medicine, 22(1), 527. | No outcome of interest |
| 38 | Zhang, Y., Song, M., Wang, M., Hertzmark, E., Wu, K., Eliassen, A. H., ... & Giovannucci, E. L. (2024). All‐cause and cause‐specific mortality risk and loss in life expectancy associated with incident type 2 diabetes onset age and duration. Journal of internal medicine, 296(3), 260-279. | No sex differences reported |
| 39 | Zhong, J., Zhang, Y., Zhu, K., Li, R., Zhou, X., Yao, P., ... & Liu, G. (2024). Associations of social determinants of health with life expectancy and future health risks among individuals with type 2 diabetes: two nationwide cohort studies in the UK and USA. The Lancet Healthy Longevity, 5(8), e542-e551. | No sex differences reported |

# **Supplementary Table 3. Cohort characteristics by multi-country studies.**

| **Tomic et al. (2022)** | |
| --- | --- |
| Australia | National Diabetes Service Scheme |
| Austria | Austrian Social Insurance |
| Canada | Canadian Chronic Disease Surveillance System |
| Denmark | National Patient Register, prescription database, health insurance database, diabetes quality database, and eye screening database |
| Finland | FinDM (Diabetes in Finland) research database |
| France | National Health Data System |
| Germany | Statutory Health Insurance claims data |
| Hong Kong | Hong Kong Hospital Authority |
| Hungary | National Institute of Health Insurance Fund Management database |
| Israel | Clalit Health Services |
| Israel | Maccabi Healthcare Services |
| Italy | Administrative health databases |
| Japan | National Database of Health Insurance Claims and Specific Health Checkups of Japan |
| Latvia | Latvian Diabetes Registry |
| Lithuania | National Compulsory Health Insurance Fund Information System |
| Netherlands | NIVEL Primary Care Database |
| Norway | Norwegian Patient Registry, Primary Care Database and Norwegian Prescription Database |
| Scotland | SCI-Diabetes database |
| Singapore | National administrative data (Ministry of Health of Singapore) |
| South Korea | National Health Insurance Service – National Sample Cohort |
| Spain | Information System for the Development of Research in Primary Care |
| Taiwan | National Health Insurance Research Database (LHID 2000) |
| United Kingdom | THIN database |
| United States | NHIS |

| **Magnussen et al. (2025)** | |
| --- | --- |
| USA | ARIC |
| USA | CARDIA |
| USA | Cardiovascular Health Study (CHS) |
| USA | Dallas Heart Study (DHS) |
| USA | Framingham Heart Study (FHS) |
| USA | Hispanic Community Health Study (HCHS/SOL) |
| USA | Jackson Heart Study |
| USA | Multi-Ethnic Study of Atherosclerosis (MESA) |
| Argentina | CESCAS |
| Argentina | RAUCH |
| Brazil | Passo Fundo |
| Brazil | EpiFloripa |
| Chile | CESCAS Chile |
| Costa Rica | Costa Rican Longevity and Healthy Aging Study (CRELES) |
| Mexico | Mexico City Diabetes Study (MCDS) |
| Mexico | Mexico City Prospective Study (MCPS) |
| Mexico | Mexican Health and Aging Study (MHAS) |
| Peru | CRONICAS |
| Peru | PERU MIGRANT Study |
| Uruguay | CESCAS Uruguay |
| Belgium | Belgian Interuniversity Research on Nutrition and Health (BIRNH) |
| Denmark | Copenhagen City Heart Study |
| Denmark | DanMONICA |
| Finland | FINRISK |
| Finland | Health 2000/2011 |
| Germany | Diabetes Cardiovascular Risk Evaluation Targets and Essential Data for Commitment of Treatment (DETECT) |
| Germany | ESTHER |
| Germany | Gutenberg Health Study (GHS) |
| Germany | Hamburg City Health Study (HCHS) |
| Germany | MONICA/KORA |
| Germany | Study of Health in Pomerania (SHIP) |
| Greece | ATTICA |
| Italy | MATISS Rome Study |
| Italy | Moli-sani |
| Italy | MONICA-Brianza |
| Italy | MONICA-Friuli |
| Italy | PAMELA study |
| Northern Ireland & France | Prospective Epidemiological Study of Myocardial Infarction (PRIME) |
| Norway | Trøndelag Health Study (HUNT) |
| Norway | Tromsø |
| Spain | MONICA Catalonia |
| Sweden | Malmö Diet and Cancer Study (MDCS) |
| Sweden | Malmö Preventive Project (MPP) |
| Sweden | Northern Sweden MONICA Cohort |
| Sweden | ULSAM |
| Sweden | Västerbotten Intervention Programme (VIP) |
| The Netherlands | Prevention of REnal and Vascular ENd stage Disease (PREVEND) |
| The Netherlands | Rotterdam Study |
| UK | British Regional Heart Study (BRHS) |
| UK | Scottish Heart Health Extended Cohort (SHHEC) |
| UK | UK Biobank |
| Russia, Poland, Lithuania and Czech Republic | Health, Alcohol and Psychosocial factors in Eastern Europe (HAPIEE) |
| Estonia | Estonian Genome Center of the University of Tartu (EGCUT) |
| Kyrgyz Republic | Interepid |
| Lithuania | Kaunas |
| Poland | Krakow |
| Poland | Warsaw |
| Russia | Novosibirsk Study |
| Russia | Interepid |
| Turkey | Turkish Adult Risk Factor Study (TARFS) |
| Gambia | Kiang West Longitudinal Study (KWLS) |
| Mauritius | Mauritian Study |
| Uganda | General Population Cohort (GPC) |
| Iran | Golestan Cohort Study (GCS) |
| Iran | Mashad Study |
| Iran | Prospective Epidemiological Research Studies in IrAN (PERSIAN) |
| Iran | Pars Cohort Study (PCS) |
| Iran | Tehran Lipid and Glucose Study (TLGS) |
| Israel | Israeli Ischaemic Heart Disease (IIHD) |
| Brunei | STEPS |
| China | China Health and Retirement Longitudinal Study (CHARLS) |
| China | Chinese Longitudinal Healthy Longevity Survey (CLHLS) |
| Japan | Hisayama Study |
| Japan | Ohsaki Cohort Study |
| Japan | Yamagata Study |
| India | Cardiometabolic Risk Reduction in South-Asia (CARRS) |
| India | Mumbai Cohort Study (MCS) |
| China | China Hypertension Epidemiology Follow-up Study (CHEFS) |
| South Korea | Namwon Study (NWS) |
| Australia | AusDiab |
| Australia | Dubbo |
| Australia | Melbourne Collaborative Cohort Study (MCCS) |
| Australia | MONICA Newcastle |
| **(Health examination survey data)** | |
| USA | National Health and Nutrition Examination Survey (NHANES) |
| Brazil | Pesquisa Nacional de Saúde (PNS) |
| Chile | Encuesta Nacional de Salud (ENS) |
| Guyana | WHO STEPS Guyana |
| Mexico | National Health and Nutrition Survey (ENSANUT) |
| Finland | National FinHealth 2017 Study |
| Germany | German Health Interview and Examination Survey for Adults (DEGS1) |
| Russia | Know Your Heart |
| Belarus | WHO STEPS Belarus |
| Georgia | WHO STEPS Georgia |
| Moldova | WHO STEPS Moldova |
| Tajikistan | WHO STEPS Tajikistan |
| Turkmenistan | WHO STEPS Turkmenistan |
| South Africa | The South African National Health and Nutrition Examination Survey (SANHANES) |
| Eswatini | WHO STEPS Eswatini |
| Ethiopia | WHO STEPS Ethiopia |
| Rwanda | WHO STEPS Rwanda |
| Sudan | WHO STEPS Sudan |
| Uganda | WHO STEPS Uganda |
| Azerbaijan | WHO STEPS Azerbaijan |
| Iraq | WHO STEPS Iraq |
| Jordan | WHO STEPS Jordan |
| Lebanon | WHO STEPS Lebanon |
| Bangladesh | WHO STEPS Bangladesh |
| Butan | WHO STEPS Butan |
| Lao | WHO STEPS Lao |
| Mongolia | WHO STEPS Mongolia |
| Myanmar | WHO STEPS Myanmar |

# **Supplementary Table 4.** Results of the Joanna Briggs Institute (JBI) Appraisal Checklist for Cohort studies.

| JBI Appraisal Checklist for Cohort studies | | | | | | | | | | | | |
| --- | --- | --- | --- | --- | --- | --- | --- | --- | --- | --- | --- | --- |
| Study | 1 | 2 | 3 | 4 | 5 | 6 | 7 | 8 | 9 | 10 | 11 | Total |
| Brown et al. (2001) | Y | Y | Y | NA | NA | Y | Y | Y | NA | NA | Y | 7/7 |
| Dhana et al. (2016) | Y | Y | Y | NA | NA | Y | Y | Y | Y | Y | Y | 9/9 |
| Goto et al. (2019) | Y | Y | Y | NA | NA | Y | Y | Y | Y | Y | Y | 9/9 |
| Hou et al. (2024) | Y | Y | Y | NA | NA | Y | Y | Y | Y | Y | Y | 9/9 |
| Huo et al. (2016) | Y | Y | Y | NA | NA | Y | Y | Y | Y | Y | Y | 9/9 |
| Laditka et al. (2015) | Y | Y | Y | NA | NA | Y | Y | Y | Y | Y | Y | 9/9 |
| Liang et al. (2020) | Y | Y | Y | NA | NA | Y | Y | Y | Y | Y | Y | 9/9 |
| Livingstone et al. (2015) | Y | Y | Y | NA | NA | Y | Y | Y | Y | Y | Y | 9/9 |
| Loukine et al. (2012) | Y | Y | Y | NA | NA | Y | Y | Y | Y | Y | Y | 9/9 |
| Magnussen et al. (2025) | Y | Y | Y | NA | NA | Y | Y | Y | Y | Y | Y | 9/9 |
| Manuel et al. (2004) | Y | Y | Y | NA | NA | Y | Y | Y | NA | NA | Y | 7/7 |
| Miller et al. (2012) | Y | Y | Y | NA | NA | Y | Y | Y | Y | Y | Y | 9/9 |
| Payne et al. (2023) | Y | Y | Y | NA | NA | Y | Y | Y | Y | Y | Y | 9/9 |
| Petrie et al. (2016) | Y | Y | Y | NA | NA | Y | Y | Y | Y | Y | Y | 9/9 |
| Preston et al. (2018) | Y | Y | Y | NA | NA | Y | Y | Y | Y | Y | Y | 9/9 |
| Price et al. (2010) | Y | Y | Y | NA | NA | Y | Y | Y | Y | Y | Y | 9/9 |
| Tachkov et al. (2020) | Y | Y | Y | NA | NA | Y | Y | Y | Y | Y | Y | 9/9 |
| Tian et al. (2024) | Y | Y | Y | NA | NA | Y | Y | Y | Y | Y | Y | 9/9 |
| Tomic et al. (2023) | Y | Y | Y | NA | NA | Y | Y | Y | Y | Y | Y | 9/9 |
| Turin et al. (2012) | Y | Y | Y | NA | NA | Y | Y | Y | NA | NA | Y | 7/7 |
| Walker et al. (2018) | Y | Y | Y | NA | NA | Y | Y | Y | Y | Y | Y | 9/9 |
| Wright et al. (2017) | Y | Y | Y | NA | NA | Y | Y | Y | Y | Y | Y | 9/9 |
| Wubishet et al. (2021) | Y | Y | Y | NA | NA | Y | Y | Y | Y | Y | Y | 9/9 |
| **Abbreviations**: N, no; NA, not applicable; NR, not reported; Y, yes.  **Items of the Joanna Briggs Institute (JBI) Appraisal Checklist for Cohort studies**:  1. Were the two groups similar and recruited from the same population?  2. Were the exposures measured similarly to assign people to both exposed and unexposed groups?  3. Was the exposure measured in a valid and reliable way?  4. Were confounding factors identified?  5. Were strategies to deal with confounding factors stated?  6. Were the groups/participants free of the outcome at the start of the study (or at the moment of exposure)?  7. Were the outcomes measured in a valid and reliable way?  8. Was the follow up time reported and sufficient to be long enough for outcomes to occur?  9. Was follow up complete, and if not, were the reasons to loss to follow up described and explored?  10. Were strategies to address incomplete follow up utilized?  11. Was appropriate statistical analysis used? | | | | | | | | | | | | |

# **Supplementary Figure 1.** Forest plot showing life expectancy estimates and its 95% confidence interval of individuals with type 1 diabetes according to sex.

# **Supplementary Figure 2.** Forest plot showing life expectancy estimates and its 95% confidence interval of individuals with type 2 diabetes according to sex.

# **Supplementary Figure 3.** Forest plot showing life expectancy estimates and its 95% confidence interval of individuals without diabetes according to sex.

# **Supplementary Figure 4.** Forest plot showing the comparison between life expectancy estimates and 95% confidence interval of men with type 1 diabetes and type 2 diabetes.

# **Supplementary Figure 5.** Forest plot showing the comparison between life expectancy estimates and 95% confidence interval of men with type 1 diabetes and without diabetes.

# **Supplementary Figure 6.** Forest plot showing the comparison between life expectancy estimates and 95% confidence interval of men with type 2 diabetes and without diabetes.

# **Supplementary Figure 7.** Forest plot showing the comparison between life expectancy estimates and 95% confidence interval of women with type 1 diabetes and type 2 diabetes.

# **Supplementary Figure 8.** Forest plot showing the comparison between life expectancy estimates and 95% confidence interval of women with type 1 diabetes and without diabetes.

# **Supplementary Figure 9.** Forest plot showing the comparison between life expectancy estimates and 95% confidence interval of women with type 2 diabetes and without diabetes.

# **Supplementary Figure 10.** Forest plot showing life expectancy estimates and its 95% confidence interval of women with type 2 diabetes according to geographical area.

# **Supplementary Figure 11.** Forest plot showing life expectancy estimates and its 95% confidence interval of men with type 2 diabetes according to geographical area.

# **Supplementary Figure 12.** Forest plot showing life expectancy estimates and its 95% confidence interval of men without diabetes according to geographical area.

# **Supplementary Figure 13.** Forest plot showing life expectancy estimates and its 95% confidence interval of women without diabetes according to geographical area.

# **Supplementary Figure 14.** Forest plot showing the years of potential life lost estimates and 95% confidence interval of individuals with type 1 diabetes.

# **Supplementary Figure 15.** Forest plot showing the years of potential life lost estimates and 95% confidence interval of individuals with type 2 diabetes.

# **Supplementary Figure 16.** Meta-regression analysis of a) cohort period and life expectancy in men with type 1 diabetes; b) cohort period and life expectancy in women with type 1 diabetes; c) cohort period and life expectancy in men with type 2 diabetes; d) cohort period and life expectancy in women with type 2 diabetes; e) cohort period and life expectancy in men without diabetes; and f) cohort period and life expectancy in women without diabetes. The solid line indicates a linear relationship. The size of each data point is proportional to its statistical weight.

a)

b)

c)

d)

e)

f)

# **Supplementary Figure 17.** LFK Index and Doi plots for publication bias detection in a) Type 1 diabetes individuals; b) Type 2 diabetes individuals; and c) Diabetes-free population.

a)

b)

c)
